# Parent Artery Occlusion Using Multiple Short iED Coils and n-Butyl Cyanoacrylate via a Marathon Microcatheter for a Dissecting Aneurysm of the Distal Posterior Inferior Cerebellar Artery With Severe Flexion of the Caudal Loop: A Case Report

**DOI:** 10.7759/cureus.84592

**Published:** 2025-05-21

**Authors:** Seigo Kimura, Daiji Ogawa, Masahiro Hayashi, Hirokatsu Taniguchi, Masahiko Wanibuchi

**Affiliations:** 1 Neurosurgery, Kouzenkai Yagi Neurosurgical Hospital, Osaka, JPN; 2 Neurology, Kouzenkai Yagi Neurosurgical Hospital, Osaka, JPN; 3 Neurosurgery and Neuroendovascular Therapy, Osaka Medical and Pharmaceutical University, Takatsuki, JPN

**Keywords:** dissecting cerebral aneurysm, endovascular coil embolization, endovascular therapy (evt), parent artery occlusion, posterior inferior cerebellar artery dissection

## Abstract

Dissecting aneurysms of the distal posterior inferior cerebellar artery (PICA) are relatively rare. This report discusses the use of multiple short iED coils via a Marathon microcatheter in a distal PICA dissecting aneurysm with severe flexion of the PICA caudal loop. A 58-year-old woman experienced a sudden, severe headache and sought consultation seven days after its onset. Head computed tomography (CT) revealed a slight left cerebellar hemorrhage. Cerebral angiography revealed a dissecting aneurysm in the telovelar tonsillar segment of the left distal PICA. Parent artery occlusion (PAO) via endovascular therapy was performed, aiming to implant coils. However, there was severe flexion of the PICA caudal loop, causing increased resistance during coil implantation, which led to the Marathon microcatheter deviating toward the basilar artery. Embolization of the dissecting aneurysm was achieved by carefully placing multiple short iED coils and administering 0.05 cc of 13% n-butyl cyanoacrylate (NBCA) via the Marathon microcatheter. Although the severe tortuosity of the PICA caudal loop made coil placement difficult, the extreme softness of the iED coil and flexibility of its delivery wire facilitated its successful placement. This case of dissecting aneurysm of the distal PICA with severe flexion of the caudal loop was successfully treated via PAO with multiple short iED coils and NBCA.

## Introduction

Distal posterior inferior cerebellar artery (PICA) aneurysms occurring beyond the bifurcation of the vertebral artery and the PICA are relatively rare, accounting for 0.38%-1.7% of all cerebral aneurysms [1,2]. Cases of ruptured distal PICA dissecting aneurysms can lead to subarachnoid hemorrhage, seen in 3.4%-58.3% of all PICA aneurysms [2]. In recent years, one of the first-line treatment options for this is endovascular treatment [3], but its associated complications and treatment strategies remain controversial [4]. Among these, the most common endovascular treatment is parent artery occlusion (PAO) using coils or liquid embolic materials such as n-butyl cyanoacrylate (NBCA) or Onyx (Medtronic, Minneapolis, MN). In performing PAO of distal PICA aneurysm, there is a risk of cerebellar infarction in the PICA region. However, because of the abundant collateral blood flow from the superior cerebellar artery or contralateral PICA, cerebellar infarction is actually unlikely to occur, and even if they do occur, they are small and unlikely to cause symptoms [5]. If complete embolization cannot be achieved using coils alone, it may be necessary to additionally use liquid embolic material as well, although liquid embolic material can flow distally from the dissecting aneurysm.

In our case, however, it was difficult to guide the microcatheter and place the coil due to severe flexion in the caudal loop of the PICA. This was addressed by using the Marathon microcatheter (Medtronic, Irvine, CA) via an intermediate catheter to place multiple short iED coils (Kaneka Medics, Osaka, Japan) and administer a small amount of NBCA, resulting in complete embolization of the dissecting aneurysm. Thus, using multiple short iED coils via the Marathon microcatheter for distal PICA dissecting aneurysms can be effective in cases with severe flexion of the caudal loop of the PICA.

## Case presentation

**Figure 1:**
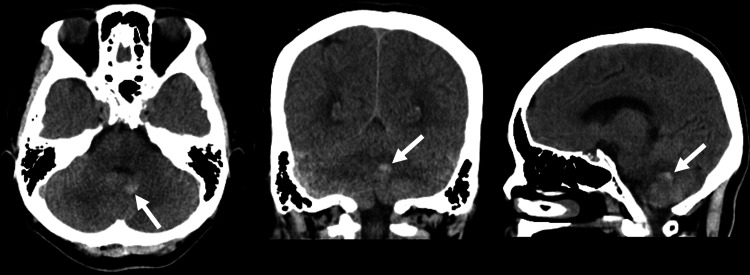
Head computed tomography revealing a slight hemorrhage (white arrow)

**Figure 2:**
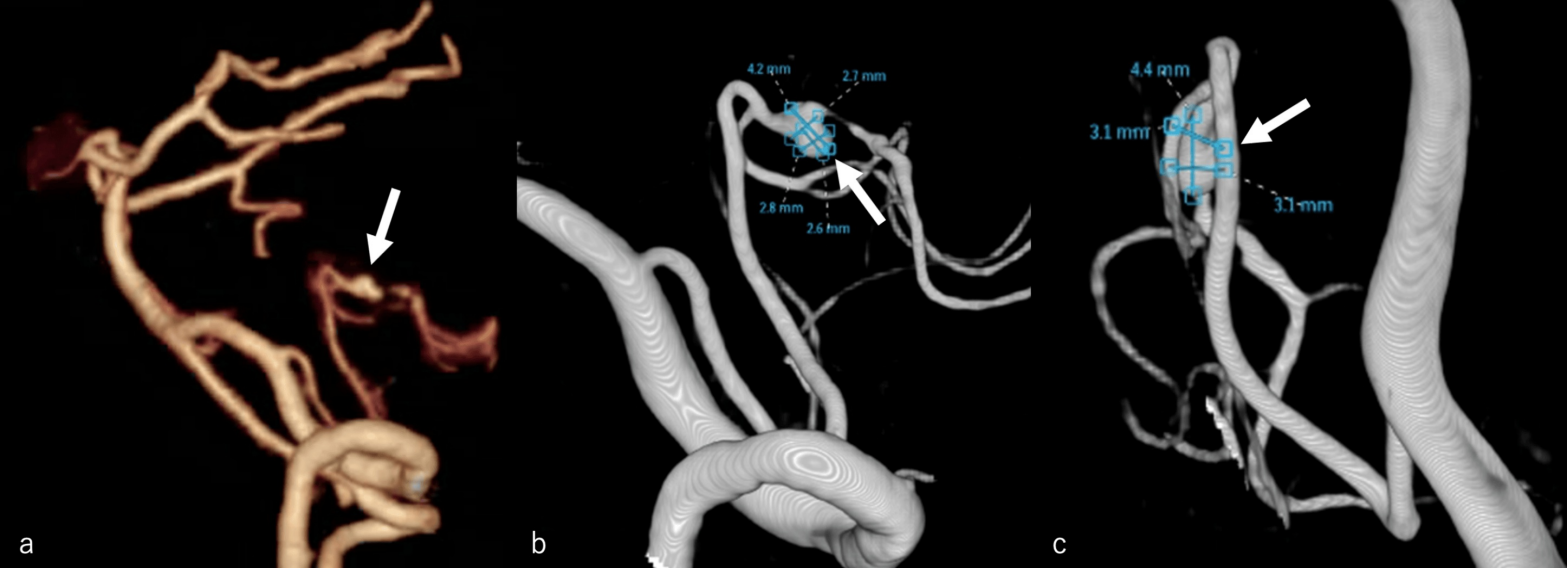
(a) Head computed tomography angiography and (b and c) cerebral angiography revealing a dissecting aneurysm in the telovelar tonsillar segment of the left distal posterior inferior cerebellar artery (b: lateral view, c: frontal view) (white arrow)

A 58-year-old woman experienced a sudden, severe headache but opted to only observe this at home. Her medical and family history were insignificant. She came to our institution seven days later, since her headache had not resolved. Upon arrival, the patient had a Glasgow Coma Scale score of 15 (E4, V5, M6), with no apparent paresis, blood pressure of 189/80 mmHg, and pulse rate of 84 beats/minute. Head computed tomography (CT) revealed a slight left cerebellar hemorrhage (Figure 1), while head CT angiography (CTA) and cerebral angiography revealed a dissecting aneurysm in the telovelar tonsillar segment (TTs) of the left distal PICA (Figure 2a-2c).

**Figure 3:**
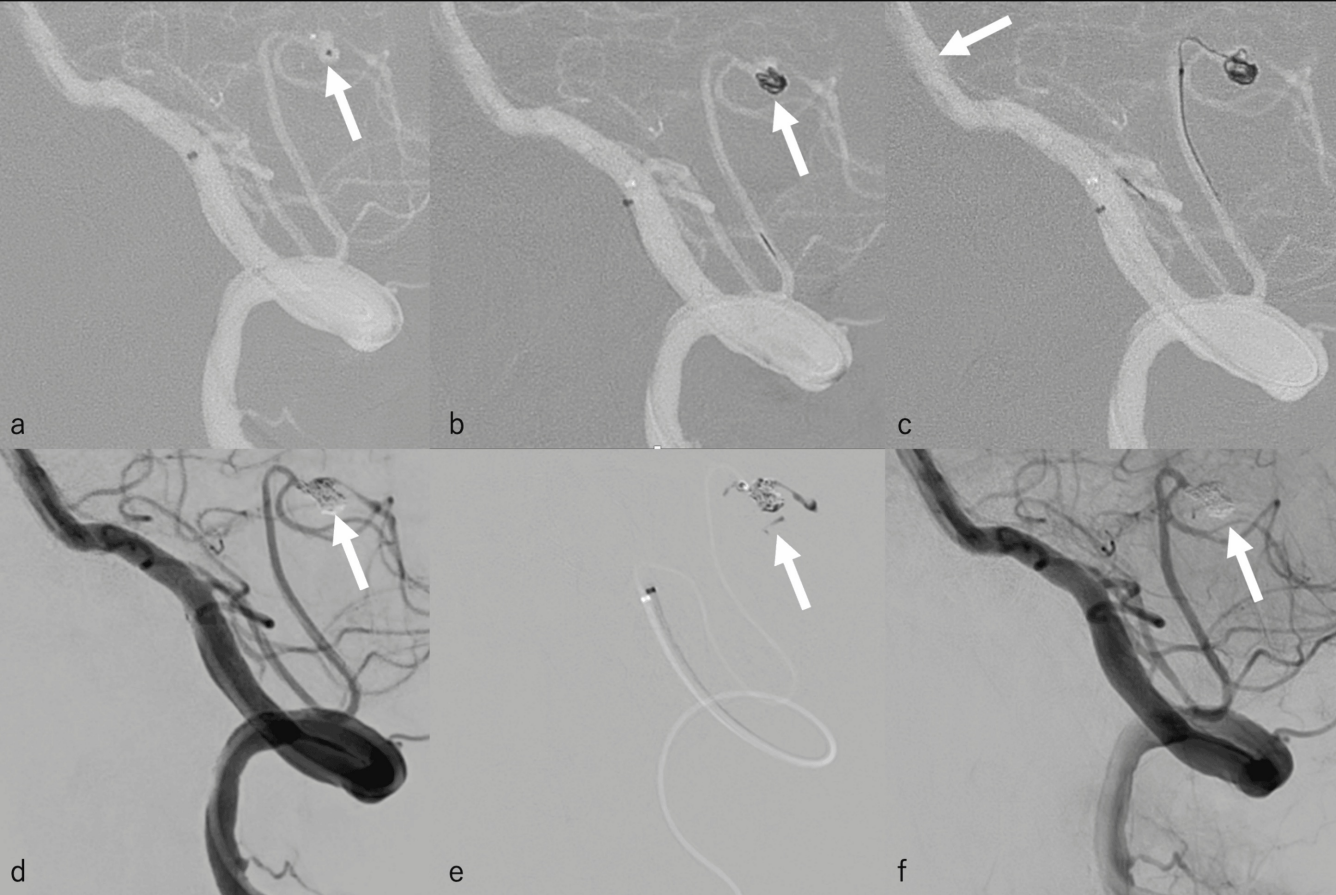
Parent artery occlusion by endovascular therapy (a) A 3.2-Fr guidepost intermediate catheter was introduced just proximal to the bifurcation of the left PICA, and the Marathon microcatheter was introduced to the dissecting aneurysm (white arrow). (b) An iED coil (Silkysoft 3 mm × 6 cm) was placed carefully as the first coil in the dissecting aneurysm (white arrow). (c) While attempting to place another coil, there was increased resistance, and the resulting force caused the Marathon microcatheter to deviate toward the basilar artery (white arrow). This was attributed to the severe flexion of the PICA caudal loop. (d) Seven smaller coils were implanted (white arrow), and (e) 0.05 cc of 13% NBCA was administered (white arrow). NBCA migrated slightly to other proximal branches but did not completely occlude. (f) Embolization was achieved, almost exclusively in the parent artery of the dissecting aneurysm (white arrow). The branch immediately proximal to the dissecting aneurysm was preserved. PICA: posterior inferior cerebellar artery, NBCA: n-butyl cyanoacrylate

PAO via endovascular therapy was performed on the same day. A 6-Fr Roadmaster guiding catheter (Goodman Co., Ltd., Aichi, Japan) was guided to the left vertebral artery (VA). Afterward, a 3.2-Fr intermediate catheter (Guidepost; Tokai Medical Products, Aichi, Japan) was introduced into the V3 segment of the left VA. The Marathon microcatheter was introduced into the dissecting aneurysm via a guidepost using a Traxcess 10 microguidewire (Microvention, Tustin, CA). We carefully attempted to place an iED Silkysoft (3 mm × 6 cm) as the first coil in the dissecting aneurysm, but the Marathon microcatheter deviated from the dissecting aneurysm as the coil passed through the caudal loop of the PICA. Therefore, the guidepost was introduced immediately proximal to the bifurcation of the left PICA, again attempting to introduce the Marathon microcatheter into the dissecting aneurysm via guidepost using Traxcess 10 (Figure 3a). The iED Silkysoft (3 mm × 6 cm) was successfully placed into the dissecting aneurysm (Figure 3b). The Marathon microcatheter has a tapered structure, and because it is a single marker, only certain coils can be used, such as the iED coil. The ED coil, which is one generation before the iED coil, is a bare platinum coil with excellent handling properties, and it is extremely soft due to the unprecedentedly small diameter of the element wire and a very flexible pusher wire system [6]. Afterward, we attempted to place an iED Silkysoft (2.5 mm × 4 cm), but this was difficult because the PICA caudal loop was severely flexed. Due to this anatomical constraint, when the resistance increased during coil implantation, force toward the basilar artery was generated, causing the Marathon microcatheter to deviate (Figure 3c). Therefore, the iED Silkysoft (2.5 mm × 4 cm) was retrieved, and instead, we attempted to implant several very short coils to avoid strong resistance and prevent the Marathon microcatheter from deviating. A total of seven coils were implanted (iED Silkysoft 3 mm × 6 cm (one coil), 2 mm × 2 cm (three coils), and 1.5 mm × 2 cm (three coils)) (Figure 3d). However, coil embolization alone did not adequately occlude the parent artery, which led us to use liquid embolic material. Using 0.05 cc of 13% NBCA (Figure 3e), the parent artery of the dissecting aneurysm was embolized almost exclusively while preserving the branch immediately proximal to the dissecting aneurysm (Figure 3f).

**Figure 4:**
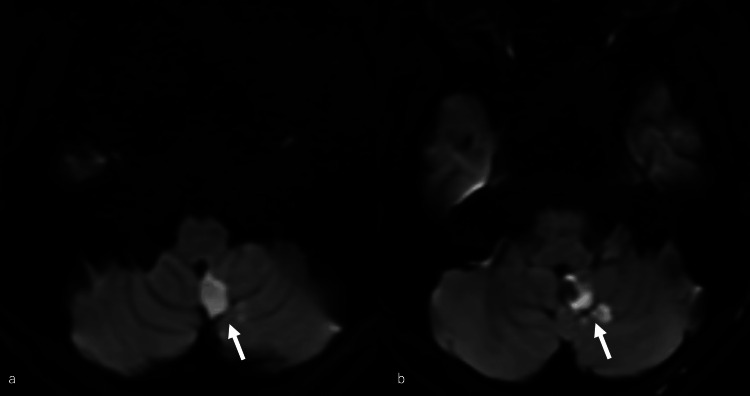
Magnetic resonance imaging performed the day after surgery revealing a cerebellar infarction (white arrow)

Magnetic resonance imaging (MRI) performed the day after surgery revealed a slight cerebellar infarction (Figure 4). The patient was dis­charged on postoperative day 33 with a modified Rankin scale of 0.

## Discussion

Distal PICA aneurysms are often saccular aneurysms proximally and dissecting aneurysms distally [2]. Due to the narrow diameter of the PICA, the typical imaging findings of a dissecting aneurysm are often not easily seen. In our case, however, head CT revealed a slight cerebellar hemorrhage, which was identified as a dissecting aneurysm upon further investigation with CTA and cerebral angiography.

The PICA is anatomically divided into five segments: the anterior medullary segment (AMs), lateral medullary segment (LMs), tonsillomedullary segment (TMs), TTs, and cortical segment (Cs) [7]. Since the vessels that perforate the brainstem may branch off from the three proximal segments (i.e., AMs, LMs, and TMs), performing PAO in these segments carries a risk of a brainstem infarction [8]. Conversely, brainstem infarction is unlikely when performing PAO in the two distal segments (i.e., TTs and Cs), but there is a risk of cerebellar infarction in the PICA region. However, this risk of cerebellar infarction can be mitigated in the presence of collateral flow from the superior cerebellar artery and contralateral PICA [5]. Because the dissecting aneurysm in our case was located in the TTs, it was unlikely that brainstem infarction would develop after PAO, making it our procedure of choice.

Distal PICA dissecting aneurysms are managed through direct surgery or neuroendovascular therapy. In particular, since ruptured distal PICA aneurysms have a higher reported re-rupture rate than other sites (e.g., supratentorial cerebral aneurysms) [9], either treatment may be desirable. The advantages of direct surgery include the direct visualization of the aneurysm and the possibility of performing hematoma removal, spinal fluid drainage, and early decompression. Conversely, neuroendovascular therapy is less invasive than direct surgery [10]. A previous report found that direct surgery and neuroendovascular therapy had comparable outcomes, although the severity of the subarachnoid hemorrhage and presence of acute hydrocephalus were associated with worse outcomes [11].

Direct surgery during cerebral vasospasm is said to increase the frequency of cerebral vasospasms. Moreover, intraoperative compression of the brain parenchyma during direct surgery can impair the cerebral circulation and worsen the prognosis [12]. In the present case, the patient sought consultation seven days after the onset of symptoms, and she was considered to be in the cerebral vasospasm phase. Although there were no apparent cerebral vasospasms at the time of admission, neuroendovascular therapy was performed after considering the possibility of inducing cerebral vasospasm during direct surgery and the degree of invasiveness. Normally, neuroendovascular therapy with stenting may be considered to preserve the parent artery. However, this patient was in the acute stage of rupture and had stenosis at the distal part of the dissection, making treatment with a stent difficult. Additionally, this stenosis may indicate a tolerance to ischemia in the distal part. For this patient, PAO was selected because of the low risk of cerebellar infarction.

The Marathon microcatheter is a liquid embolic delivery catheter that is often used for arteriovenous malformation (AVM) embolization in combination with the liquid agent NBCA or Onyx [6]. It has a smaller, 1.5-Fr distal tip (0.013 inch), which enables further navigation to the intracranial arteries. In the present case, the PICA caudal loop was severely tortuous, and the dissecting aneurysm was located distally in the cranial loop. Thus, using a long, thin microcatheter was thought to be appropriate. We also considered performing PAO in combination with NBCA and used Marathon for the endovascular therapy. If PAO was performed with NBCA alone, the NBCA would migrate to the distal portion of the dissecting aneurysm. Therefore, PAO was performed with the coil as much as possible, and NBCA was used in combination.

The Marathon microcatheter has a tapered structure, and because it is a single marker, only certain coils can be used, such as the iED coil. Because the distance was not too far in this case, it was possible to choose a catheter other than the Marathon microcatheter, which would have made it possible to use other types of coils. The ED coil, which is one generation before the iED coil, is a bare platinum coil with excellent handling properties, and it is extremely soft due to the unprecedentedly small diameter of the element wire and a very flexible pusher wire system [6]. Another study reports that the delivery wire tip of the ED coil had the highest flexibility compared to other coils [13]. Furthermore, the Kaneka Medix Corporation states that the tip of the delivery wire has become even more flexible now that iED coils are used. In our case, it was difficult to place the first coil due to the severely tortuous caudal loop of the PICA, but this was successfully accomplished by maneuvering the guidepost to the bifurcation of the PICA. However, while inserting the second coil, the delivery wire was stiff with some resistance, likely due to the severe tortuosity. This caused the Marathon microcatheter to deviate toward the basilar artery. Nevertheless, by using the shortest coils possible and slowly applying force, seven coils were eventually placed. The severe tortuosity of the PICA caudal loop made coil placement extremely difficult, but the extreme softness of the iED coil and flexibility of its delivery wire made it possible to place. Thus, this technique, using the Marathon microcatheter and iED coil, may be suitable for treating aneurysms that occur in relatively distal and tortuous arteries, such as in this case.

In a previous report, a 3.4-Fr Tactics intermediate catheter (Technocrat Corporation, Aichi, Japan) was inserted into the orifice of the PICA, and the DeFrictor Nano Catheter (Medicos-Hirata, Osaka, Japan) was guided as distally as possible to administer NBCA [14]. However, even if an intermediate catheter is already guided into the orifice of the PICA, this should still be manipulated carefully.

In the present case, we administered 0.05 cc of 13% NBCA. At our institution, we mainly use 13% NBCA, so we used the same concentration in the present case. Increasing the concentration of NBCA makes it possible to embolize with a small amount, but there is a risk that the marathon will be stuck. In this case, coil placement was difficult in this situation, but we were able to place the coil as far as possible and embolize the dissecting aneurysm almost exclusively without massive migration of NBCA. Thus, PAO using multiple short iED coils and NBCA delivered via the Marathon microcatheter is a feasible and effective approach to dissecting aneurysms of the distal PICA with severe flexion of the caudal loop. 

## Conclusions

PAO via endovascular therapy, using multiple iED coils alongside NBCA administration, was successfully performed for a case of dissecting aneurysm of the distal PICA with severe flexion of the caudal loop. PAO may cause stroke, so it is not a first-tier therapy, but a rescue one. Notably, the severe tortuosity of the caudal loop can cause instability during catheter manipulation or coil placement. This report highlights the feasibility and effectiveness of this approach for similar cases.

## References

[1] Horiuchi T, Tanaka Y, Hongo K, Nitta J, Kusano Y, Kobayashi S (2003). Characteristics of distal posteroinferior cerebellar artery aneurysms. Neurosurgery.

[2] Lewis SB, Chang DJ, Peace DA, Lafrentz PJ, Day AL (2002). Distal posterior inferior cerebellar artery aneurysms: clinical features and management. J Neurosurg.

[3] Bradac GB, Bergui M (2004). Endovascular treatment of the posterior inferior cerebellar artery aneurysms. Neuroradiology.

[4] Tang J, Wei L, Li L (2016). Endovascular treatment of distal posterior inferior cerebellar artery aneurysms. Neurosciences (Riyadh).

[5] Chalouhi N, Jabbour P, Starke RM (2013). Endovascular treatment of proximal and distal posterior inferior cerebellar artery aneurysms. J Neurosurg.

[6] Horie N, Hayashi K, Morikawa M, Izumo T, Nagata I (2015). A novel method for super-selective coil embolization using an extremely soft bare coil through a liquid embolic delivery microcatheter. Neurol Med Chir (Tokyo).

[7] Lister JR, Rhoton AL Jr, Matsushima T, Peace DA (1982). Microsurgical anatomy of the posterior inferior cerebellar artery. Neurosurgery.

[8] Ioannidis I, Nasis N, Andreou A (2012). Endovascular treatment of ruptured dissecting posterior inferior cerebellar artery aneurysms. Interv Neuroradiol.

[9] Sejkorová A, Cihlář F, Hejčl A, Lodin J, Vachata P, Sameš M (2016). Microsurgery and endovascular treatment of posterior inferior cerebellar artery aneurysms. Neurosurg Rev.

[10] Bacigaluppi S, Bergui M, Crobeddu E, Garbossa D, Ducati A, Fontanella M (2013). Aneurysms of the medullary segments of the posterior-inferior cerebellar artery: considerations on treatment strategy and clinical outcome. Neurol Sci.

[11] Hong YH, Kim CH, Che GS, Lee SH, Ghang CG, Choi YS (2011). Predicting factors affecting clinical outcomes for saccular aneurysms of posterior inferior cerebellar artery with subarachnoid hemorrhage. J Korean Neurosurg Soc.

[12] Haley EC Jr, Kassell NF, Torner JC (1992). The international cooperative study on the timing of aneurysm surgery. The North American experience. Stroke.

[13] Koyama J, Hanaoka Y, Sato A (2014). Characteristic features of coil delivery wires for cerebral aneurysm embolization. J Neuroendovasc Ther.

[14] Shintoku R, Marushima A, Okune S (2023). Endovascular embolization with n-butyl cyanoacrylate for ruptured distal posterior inferior cerebellar artery dissecting aneurysm. Asian J Neurosurg.

